# SOX9 Regulates Multiple Genes in Chondrocytes, Including Genes Encoding ECM Proteins, ECM Modification Enzymes, Receptors, and Transporters

**DOI:** 10.1371/journal.pone.0107577

**Published:** 2014-09-17

**Authors:** Chun-do Oh, Yue Lu, Shoudan Liang, Yuko Mori-Akiyama, Di Chen, Benoit de Crombrugghe, Hideyo Yasuda

**Affiliations:** 1 Department of Genetics, The University of Texas M. D. Anderson Cancer Center, Houston, Texas, United States of America; 2 Department of Bioinformatics and Computational Science, The University of Texas M. D. Anderson Cancer Center, Houston, Texas, United States of America; 3 Department of Pathology and Immunology, Baylor College of Medicine, Houston, Texas, United States of America; 4 Department of Biochemistry, Rush University Medical Center, Chicago, Illinois, United States of America; Hokkaido University, Japan

## Abstract

The transcription factor SOX9 plays an essential role in determining the fate of several cell types and is a master factor in regulation of chondrocyte development. Our aim was to determine which genes in the genome of chondrocytes are either directly or indirectly controlled by SOX9. We used RNA-Seq to identify genes whose expression levels were affected by SOX9 and used SOX9 ChIP-Seq to identify those genes that harbor SOX9-interaction sites. For RNA-Seq, the RNA expression profile of primary *Sox9^flox/flox^* mouse chondrocytes infected with Ad-CMV-*Cre* was compared with that of the same cells infected with a control adenovirus. Analysis of RNA-Seq data indicated that, when the levels of *Sox9* mRNA were decreased more than 8-fold by infection with Ad-CMV-*Cre*, 196 genes showed a decrease in expression of at least 4-fold. These included many cartilage extracellular matrix (ECM) genes and a number of genes for ECM modification enzymes (transferases), membrane receptors, transporters, and others. In ChIP-Seq, 75% of the SOX9-interaction sites had a canonical inverted repeat motif within 100 bp of the top of the peak. SOX9-interaction sites were found in 55% of the genes whose expression was decreased more than 8-fold in SOX9-depleted cells and in somewhat fewer of the genes whose expression was reduced more than 4-fold, suggesting that these are direct targets of SOX9. The combination of RNA-Seq and ChIP-Seq has provided a fuller understanding of the SOX9-controlled genetic program of chondrocytes.

## Introduction

The multistep differentiation process of chondrogenesis has an essential role in the development of the endochondral skeleton. Mesenchymal progenitor cells first form chondrogenic condensations and then differentiate into overt chondrocytes characterized by a high level of expression of a number of typical cartilage extracellular matrix (ECM) genes. These cells then sustain additional changes: first, a largely unidirectional proliferation step, followed by exit from the cell cycle and further differentiation into prehypertrophic and hypertrophic chondrocytes. These steps result in formation of the characteristic features of the so-called growth plate of endochondral bones.

Several lines of evidence indicate that the transcription factor SOX9 acts as a master regulatory factor in this multistep pathway of chondrocyte differentiation [1], [2]. Heterozygous mutations in *Sox9* cause campomelic dysplasia, a generalized and severe disease of cartilage characterized by hypoplasia of endochondral bone [3], [4]. A related disease called Pierre Robin sequence, which mainly affects the craniofacial skeleton, is associated with mutations in conserved DNA elements on either side of the *Sox9* gene [5]. Conditional inactivation of the *Sox9* gene at various times during mouse limb development demonstrated that SOX9 is necessary for mesenchymal condensations and for the commitment of the cells in these condensations to the chondrocytic cell fate.

SOX9 activates a number of genes expressed in chondrocytes, including typical cartilage ECM genes *Col2a1*, *Col9a1*, *Col11a2*, *Acan*, *CD-rap*, and others [6]–[8]. Two other SOX family members, SOX5 and SOX6, have major roles in establishing the high level of expression of several of these genes. Previous studies also showed that, during development, SOX9 was needed for the expression of *Sox5* and *Sox6* [2]. In a mouse model that phenocopies the generalized cartilage hypoplasia of campomelic dysplasia, hypertrophic chondrocyte differentiation is accelerated, suggesting that SOX9 has a role in this differentiation step.

A number of signaling pathways control either the expression or the activity of SOX9 in chondrocytes. Bmp signaling is needed for formation of chondrogenic mesenchymal condensations and for expression or maintenance of *Sox9* RNA at this step [9]. Interleukin-1 and tumor necrosis factor alpha inhibit *Sox9* expression, whereas fibroblast growth factor (FGF) upregulates *Sox9* through mitogen-activated protein kinase signaling. Parathyroid hormone-related protein in the growth plate of endochondral bones increases SOX9 activity, whereas Wnt/β-catenin inhibits SOX9 activity [10]–[14].

To better understand the SOX9-regulated genetic program of chondrocytes, we have used genome-wide methods to identify the genes that are controlled by SOX9 in these cells. To increase the specificity of these experiments, we limited our analysis, as much as was possible, to chondrocytes and examination of the effects of deletion of *Sox9* in these cells. To this end, we used primary rib chondrocytes isolated from *Sox9^flox/flox^* mice in short-time culture [2]. These cells were infected with adenoviral vector Ad-CMV-*Cre* to specifically remove the *Sox9* gene. By using the genome-wide RNA-Seq method, the RNA profile of these cells was then compared to that of the same primary chondrocytes infected with a control adenovirus [15].

Although a number of genes, mainly ECM genes, have been previously shown to be regulated by SOX9, an understanding of the broader genomic program of SOX9-dependent chondrocyte differentiation requires identification of the large spectrum of genes that are regulated by SOX9. It is also important to discriminate the genes that are directly regulated from those that are indirectly regulated by this transcription factor. To identify all the direct targets of SOX9 in the genome of chondrocytes, we used chromatin immunoprecipitation sequencing (ChIP-Seq), a method for genome-wide profiling of DNA binding and chromatin proteins, which offers higher resolution and greater coverage than its array-based predecessor ChIP on chip array [16], [17]. This tool is thought to be essential for a better understanding of transcriptional control in chondrocytes. We proposed that the combination of RNA-Seq and ChIP-Seq should provide a fuller understanding of the SOX9-controlled genetic program of chondrocytes than other methods.

## Results and Discussion

### Genes regulated by SOX9 in chondrocytes

**Figure 1:**
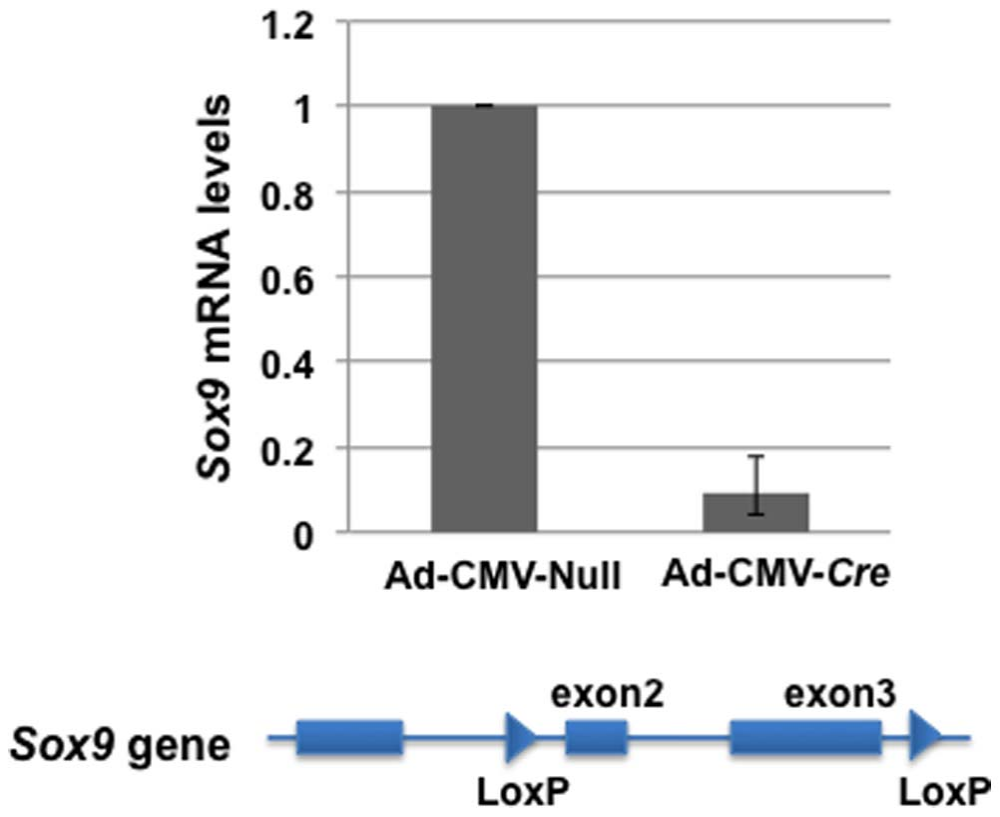
Decrease of *Sox9* expression in mouse *Sox9^flox/flox^* primary rib chondrocytes infected with Ad-CMV*-Cre*. Primary rib chondrocytes obtained from 4-day-old *Sox9^flox/flox^* mice were cultured for 24 hours and then infected with Ad-CMV*-Cre* or control vector Ad-CMV-Null at 200 moi. In these cells, exon 2 through exon 3 of the *Sox9* gene was flanked with *loxP* sites. Forty-eight hours after infection, the *Sox9* mRNA levels in cells infected with Ad-CMV-Cre, measured by RT-qPCR, were decreased by more than 8-fold compared to the levels in cells infected with Ad-CMV-Null.

Rib chondrocytes were isolated from 4-day-old *Sox9^flox/flox^* mice, in which the DNA segment containing exons 2 and 3 of the *Sox9* gene is flanked by *loxP* sites (i.e., floxed) [2]. After 1 day of culture, the semi-confluent cells were infected with Ad-CMV*-Cre* or Ad-CMV-Null virus. Forty-eight hours after infection, the cells were harvested and the expression of *Sox9* in total RNA was determined by reverse-transcription quantitative polymerase chain reaction (RT-qPCR). The CMV-driven Cre-recombinase deleted the DNA segment covering the two floxed exons of *Sox9*, resulting in a major decrease of SOX9-dependent transcription in these cells. The expression level of *Sox9* in cells infected with the Ad-CMV*-Cre* virus at 200 moi was approximately 8- to 10-fold less than that of the cells infected with control virus (Figure 1). The polyA-containing RNAs of these cells were transcribed into cDNA by use of oligo dT and, after adaptor ligation to the cDNA, a library was prepared for second-generation sequencing that proceeded from both ends of the cDNAs. RNA-Seq data were aligned to the mouse genome sequence, and the tag number showed the number of alignments. The highest tag number was 158,000 for the *Col2a1* gene in the cells infected with Ad-CMV-Null virus. The expression level of the β*-actin* gene was used as a normalization control in both samples, because its expression levels as determined by qPCR in the cDNAs used for RNA-Seq were similar in both cells.

Genes used for the analysis had a z-score higher than 4.0 and a level of expression that was more than 0.06% (100 tags) of the expression level of *Col2a1* in cells infected with Ad-CMV-Null virus. The numbers of genes whose expression was either decreased or increased more than 2-fold by SOX9 depletion were 1,400 and 65, respectively (Table 1). This result suggests that SOX9 is mainly an activator of transcription in chondrocytes. The numbers of genes that showed a decrease in expression of more than 8-, 6-, or 4-fold were 45, 83, and 196, respectively. In contrast, the numbers of genes whose expression was increased by more than 8-, 6-, or 4-fold were 6, 8, and 16, respectively (Table 1).

**Table 1:** The % of genes regulated by SOX9, that have SOX9 binding sites.

	Results from RNA-Seq	Genes that have SOX9 binding sites (mouse)	Genes that have SOX9 binding sites (rat)
RNA	No. of genes	Total 1717	Total 1133
> = 2 decrease	1400	251 (17.9%)	176 (12.6%)
> = 3 decrease	384	114 (29.7%)	87 (22.7%)
> = 4 decrease	196	71 (36.2%)	62 (31.6%)
> = 6 decrease	83	39 (47%)	36 (43.3%)
> = 8 decrease	45	25 (55.6%)	23 (51.1%)
> = 2 increase	65	4 (6.2%)	2 (3.1%)
> = 3 increase	26	4 (15.4%)	1 (3.8%)
> = 4 increase	16	2 (12.5%)	1 (6.3%)
> = 6 increase	8	0	0
> = 8 increase	6	0	0

**Figure 2:**
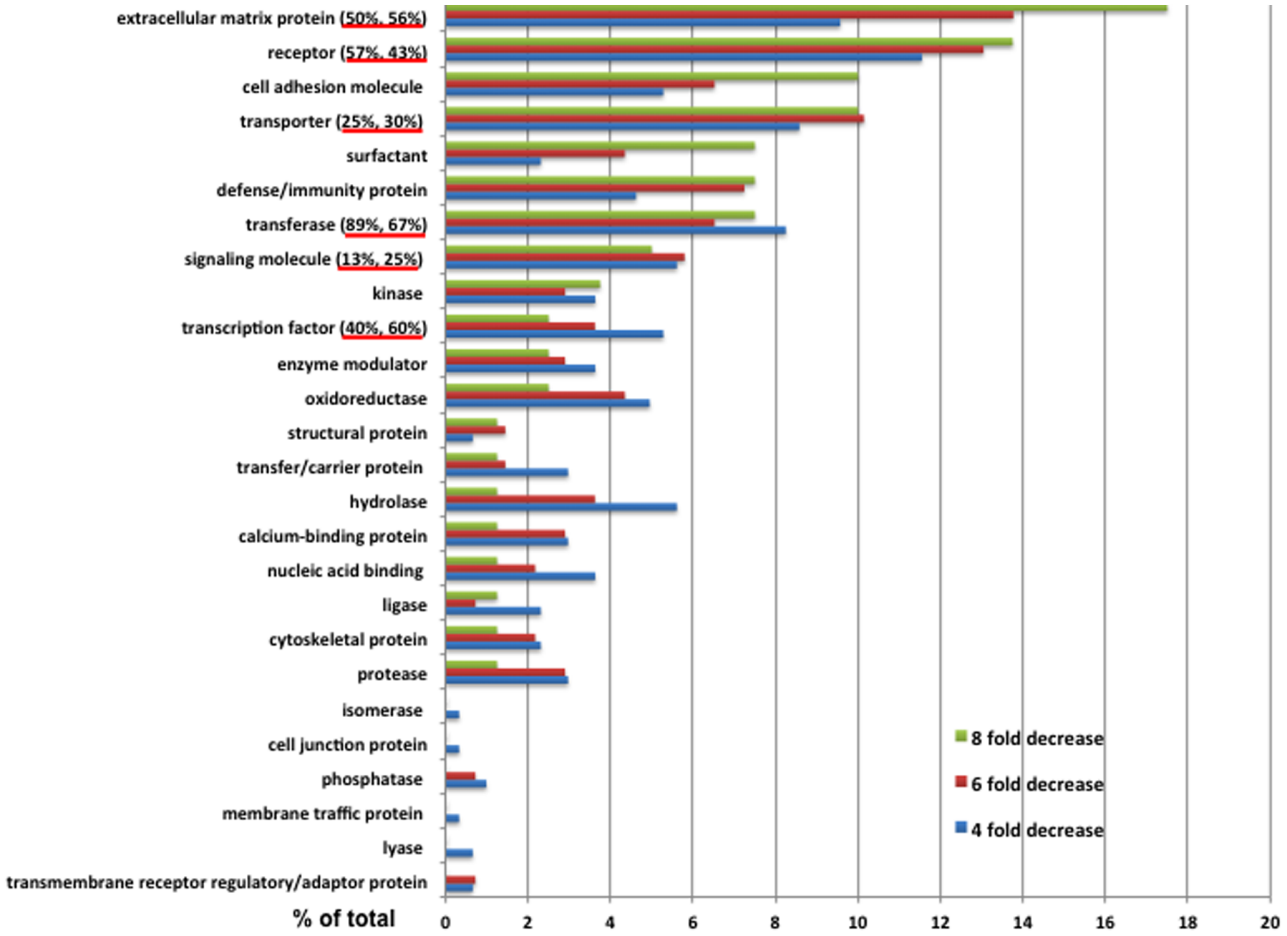
Classification of genes whose expression was decreased after removal of SOX9. Genes whose expression was decreased by more than 4-, 6-, or 8-fold by the removal of SOX9 were classified into functional categories. The percentage of proteins in each category is shown. The number of corresponding proteins in each category is shown in Table S1. Some proteins were assigned to more than one category. Genes whose expression was decreased by more than 6-fold by the removal of SOX9 were classified into 20 functional groups; the percentages in each group of more than five genes containing a SOX9-interaction site revealed by ChIP-Seq are underlined in red on the left: the first percentage is for mouse rib chondrocytes and the second for RCS cells. Among the genes whose expression was decreased by more than 6- or 8-fold, the ECM and transferase groups had the highest percentages of SOX9-interaction sites, and these percentages were higher than for the same groups of genes whose expression was decreased by only 4-fold after SOX9 removal.

The genes whose expression was decreased by more than 8-, 6-, or 4-fold after deletion of *Sox9* were classified according to a Panther gene classification system (http://www.pantherdb.org), which orders genes according to the functional characteristics of the corresponding proteins. The protein categories of the genes whose expression was decreased by more than 8-, 6-, or 4-fold are shown in Figure 2 and Table S1. Some genes were assigned to more than one category by the Panther program. The greatest number of genes were linked to ECM proteins, the next greatest to transferases, and so on to receptors, transporters, hydrolases, transcription factors, and signaling molecules. The proportion of genes in the ECM and transferase groups markedly increased when the genes whose expression was decreased by 6- or 8-fold were compared to genes decreased by 4-fold. The ECM proteins represented included several collagen types, aggrecan, matrilin-3, and others, whereas the transferase group included exostosin-like 1, xylosyltransferase,N-acetyl-beta-glucosaminyl-glycoprotein, and 4-beta-N-actylgalactosminyltransferase. Among the transporter proteins were potassium voltage-gated channel subfamily S member 1 (KCNS1) and transient receptor potential cation channel subfamily V member 4 (TRPV4). The group of receptors included FGF receptor 3 (FGFR3).

Since Sox9 is a master factor regulating chondrocyte differentiation, the deficiencies and/or mutations of the proteins regulated by Sox9 could be the cause of skeletal diseases. ECM proteins serve many important functions that provide characteristic properties to cartilage tissues, including the regulation of intercellular communication. The expressions of several ECM genes, including those for aggrecan (*Acan*) and collagens II, IX, and XI, were previously shown to be regulated by SOX9. A number of mutations in ECM proteins are associated with skeletal diseases (Table S3). Mutations in genes for proteins other than ECM proteins that appear to be regulated by SOX9 are also known to be associated with skeletal diseases (Table S3). For instance, mutations in exostosin-like 1, an enzyme that modifies ECM proteins, are associated with skeletal malfunction [18]. The transferases represented by these genes function mainly in posttranslational modifications of a number of ECM proteins, thereby completing the synthesis of cartilage ECM proteins. We speculate that ion transporters are also likely to have an important role in forming and maintaining the particular nature of cartilage. Among other genes that were markedly downregulated by SOX deletion is *fibin*, which has been shown recently to regulate fin development of the zebrafish skeleton [19]. Several signaling molecules are known to have important roles in the regulation of chondrocyte differentiation, namely FGFs, Wnts, platelet-derived growth factor (PDGF), integrins, and molecules involved in inflammation and in transforming growth factor beta (TGFβ) signaling. In humans, activating mutations in FGFR3 are associated with several forms of dwarfism, including achondroplasia, thanatophoric dysplasia, and hypochondroplasia. Mutations in the transmembrane domain of FGFR3 cause the most common genetic form of dwarfism, achondroplasia [20], probably because FGFR3 controls the proliferation of chondrocytes and has been shown to stimulate SOX9 expression [10]. Together with the finding that Sox9 in turn also activates FGFR expression this represents a possible positive feedback loop [21]. The genes for Wnt antagonists FZD9 and FRZB [22]–[24] were downregulated more than 10-fold, whereas the gene for LRP4 [25], which acts as a negative regulator of Wnt signaling, was also downregulated. The inhibition of Wnt signaling is important for chondrocyte differentiation when chondrogenic and osteogenic precursors segregate from a common progenitor. Wnt signaling competes with and inhibits the function of SOX9 [26]. The integrin, PDGF, and TGFβ signaling are thought to be also important for chondrocyte proliferation and differentiation [27]–[29].

In contrast to the genes whose expression was decreased following ablation of *Sox9* in chondrocytes, genes with a 4-fold increase in expression did not fall into a specific functional group, except for a very small number of genes classified as coding for cell adhesion molecules, for ECM proteins, for receptors, or for proteases (Table S2 and Figure S1). The genes with increased expression by more than 8-fold after removal of Sox9 are listed (Table S4).

### Genes that have SOX9-interaction sites in chondrocytes

Our RNA-Seq results strongly suggested that, in chondrocytes, many genes coding for proteins classified in several specific functional groups are regulated by SOX9. Previous results from both reporter assays and transgenic mice studies had provided evidence that only a few cartilage ECM genes, including *Agc1*, *Col2a1*, *Col11a2*, *CD-rap*, and a few others are directly regulated by SOX9 in chondrocytes [6]–[8], [30]. We therefore decided to identify which genes in chondrocytes contained SOX9-interaction sites and should be considered strong candidates to be direct targets of SOX9.

### SOX9 Chip-Seq

To identify the SOX9-interaction sites in the genome of chondrocytes, we performed ChIP-Seq experiments using both primary mouse rib chondrocytes, which were essentially the same cells as those used in the RNA-Seq experiment, and rat chondrosarcoma (RCS) cells, which retain many specific characteristics of chondrocytes [31], [32]. For both cells we used the criterion that peaks should be more than 2-fold higher than the input background. Using this criterion, we identified 2,364 peaks associated with 1,742 genes with a p value of less than 10^−5^ in rib chondrocytes and 3,254 peaks associated with 1,978 genes with a p value of less than 10^−8^ in RCS cells. A total of 377 peaks and 638 peaks were outside the genes in rib chondrocytes and RCS cells, respectively, and some genes contained two or more peaks. The number of genes containing peaks was comparable to the ∼2,000 genes that had been shown to be regulated by SOX9 more than 2-fold over controls in the RNA-Seq experiments.

**Figure 3:**
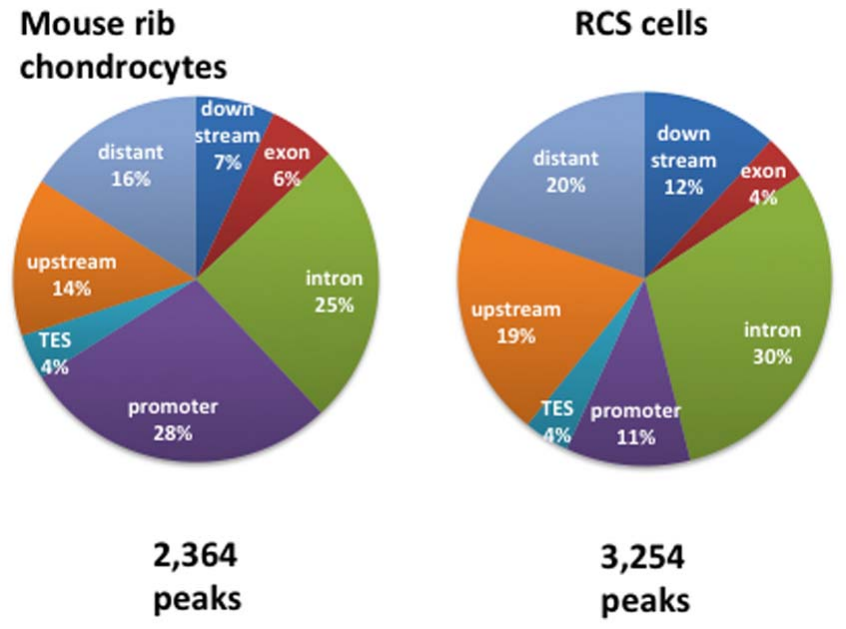
Distribution of SOX9-interacting peak locations. The locations are defined as follows: upstream: −50 to −5 Kb from transcription start site (TSS); promoter: −5 to +0.5 Kb from TSS; termination site (TES): −0.5 to +5 Kb from TES; downstream: +5 to +50 Kb from TES; distant: localized outside the boundaries already defined. Both mouse and rat genes were aligned by using the database of mouse genome sequences because the annotation and the assignment of genes to the rat genome is not yet complete.

The distribution of peaks in and around these genes is shown in Figure 3. In both cell types the number of peaks was high in introns. In rib chondrocytes, the number in promoter regions, defined as the segment between +1 (the transcription start site) and −5 Kb, was also high. In each gene category identified by RNA-Seq, the percentage of genes whose expression was decreased by more than 6-fold that contained a SOX9-interaction site(s) is shown in Figure 2: in each case, the two numbers correspond to mouse rib chondrocytes and RCS cells, respectively.

**Figure 4:**
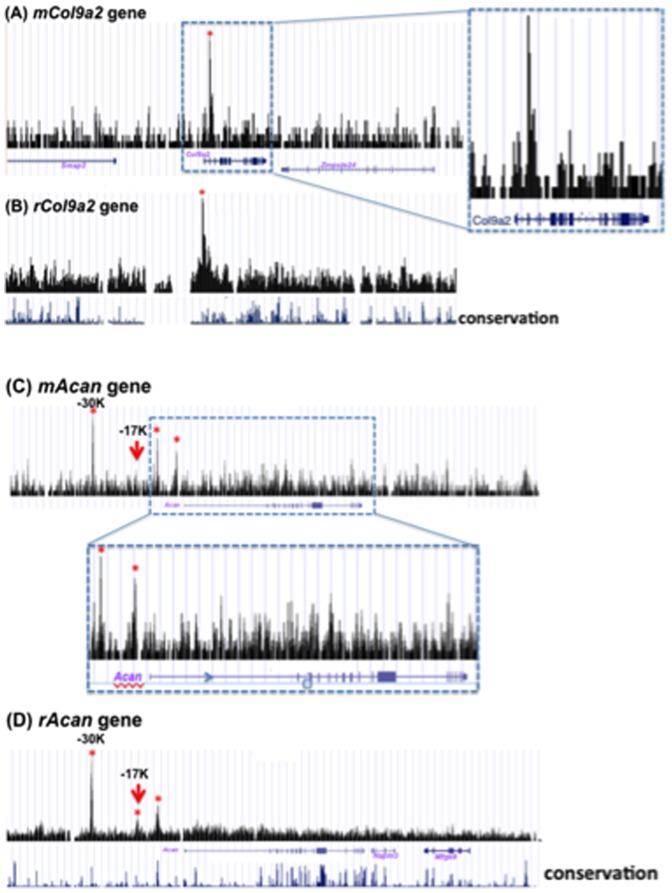
Examples of ChIP-Seq peaks of mouse primary rib chondrocytes and RCS cells. The SOX9-interacting peaks of the *Col9a2* (collagen IX, alpha-2 polypeptide) and *Acan* (aggrecan) genes are shown in A, B) and C, D), respectively. In the bottoms of B) and D), the sequence conservation peaks among 6 vertebrates are shown. The peaks detected by our criteria are indicated by *. A) The peaks in the mouse *Col9a2* gene from −5 Kb from the TSS to +5 Kb from the TSE are shown. In the right of this graph, the portion surrounded by dotted square is shown. The solid squares in *Col9a2* gene show the exons including non-coding ones. B) The peaks in the rat *Col9a2* gene are shown. In this region two additional genes, *Zmpste24* and *Smap2*, exist in addition to *Col9a2*. C) The peaks in the mouse *Acan* gene from −50 Kb from the TSS to +50 Kb from the TSE are shown. Below in this graph, the portion surrounded by dotted square in C) is shown. D) The peaks in the rat *Acan* gene are shown. The solid squares in *Acan* gene show the exons including non-coding ones. In this region, two additional genes in addition to *Acan*, *Hapln3* and *Mfge8*, are localized in the reverse direction to *Acan*. Three sharp peaks were detected in mouse and rat *Acan* genes. The locations of two of the three peaks are similar in genes of mouse chondrocytes and rat RCS cells. The red arrows show the peaks that have been shown to have SOX9/SOX5/SOX6-dependent enhancer activity [33].

**Figure 5:**
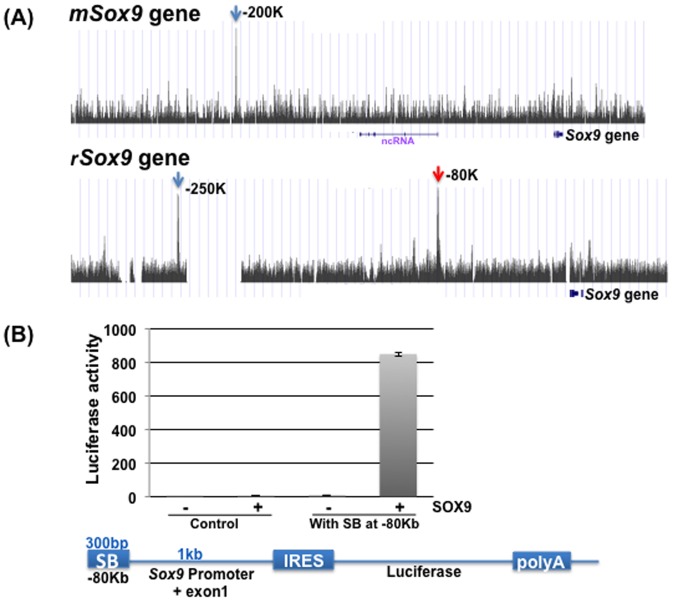
The SOX9-dependent enhancer activity of the SOX9-interacting peak at −80 Kb in the *Sox9* gene. A) The peaks in the *Sox9* gene from −300 Kb from the TSS to +50 Kb from the TSE are shown. In this region, no additional coding genes exist except for a mouse non-coding RNA in the reverse direction to *Sox9*. One peak (blue arrow) was detected in the mouse *Sox9* gene and two sharp peaks (blue and red arrows) were observed in the rat *Sox9* gene. B) The SOX9-dependent enhancer activity of the −80 Kb peak in the rat gene (red arrow) is demonstrated. A 750-bp DNA fragment surrounding the SOX9-interacting site at −80 Kb from the TSS was inserted 5′ to a 1-Kb *Sox9* promoter plus exon1 fragment followed by an internal ribosome entry site (IRES) and a luciferase reporter as illustrated at the bottom of the figure. 293T cells were transfected with either a control vector or this construct in the presence or absence of a *Sox9*-expressing plasmid, and the luciferase activity was measured 48 hours after transfection. The reporter activity, which was normalized with *renilla* luciferase activity, was markedly increased in the presence of SOX9.

Characteristic ChIP-Seq patterns for *Acan*, *Col9a2*, and *Sox9* in the genomes of rib chondrocytes and RCS cells are shown in Figures 4 and 5. The conservation graphs in the figures were obtained by the vertebrate multi alignment & conservation program of the UCSC genome browser (https://genome.ucsc.edu/cgi-bin/hgGateway). In this program, the genome sequences of mouse, human, dog, opossum, chicken, and x. tropicalis were used. The peaks are located at sites where sequence conservation scores were high. Using our criteria that peaks should be more than 2-fold higher than the input background, in *Col9a2*, there was one sharp interaction site and minor sites in intron 1 in both cell types. The *Acan* gene had three peaks in RCS cells and in rib chondrocytes (marked by * in Figure 4). The two interaction sites were in very similar positions in the two species. One of the SOX9-interaction sites located at ∼17 Kb upstream of the transcription start site of the *Acan* gene in RCS cells is identical to the site shown to have SOX9-dependent enhancer activity in the presence of SOX5 or SOX6 (Figure 4, red arrow) [33]. The *Sox*9 gene had two sites in RCS cells. One of these sites is located −80 Kb upstream of the *Sox9* gene and the other is −250 Kb upstream from the transcription start site (Figure 5A, shown by red and blue arrow, respectively). The conservation of the sequences upstream is not always retained between mouse and rat, whereas the sequence of the peak (blue arrow) located at −200 Kb in rib chondrocytes is very similar to that of the peak (blue arrow) located at −250 Kb in RCS cells (Figure S2). To verify whether this site is a SOX9-dependent enhancer, the 750-bp DNA fragment surrounding the peak at −80 Kb (Figure 5A, red arrow) was inserted into a luciferase reporter containing a 850-bp *Sox9* promoter fragment. The activity of this reporter in 293T cells was very strongly enhanced in the presence of SOX9 (Figure 5B), indicating that this site has strong SOX9-dependent enhancer activity. A similar reporter assay was performed with the DNA fragment surrounding the peak around −250 Kb from the transcription start site of *Sox9*. Enhancement of activity, to a lesser degree than with the −80 Kb peak, was seen in the presence of both SOX9 and SOX5 (data not shown). Note that sites located more than −50 Kb upstream of the genes were counted as distant sites in Figure 3. However, these data suggest that functional SOX9 interactions exist even in far upstream segments and that the sites identified by ChIP-Seq experiments may represent physiologically relevant SOX9-interaction sites.

### Search for a SOX9-binding motif

**Figure 6:**
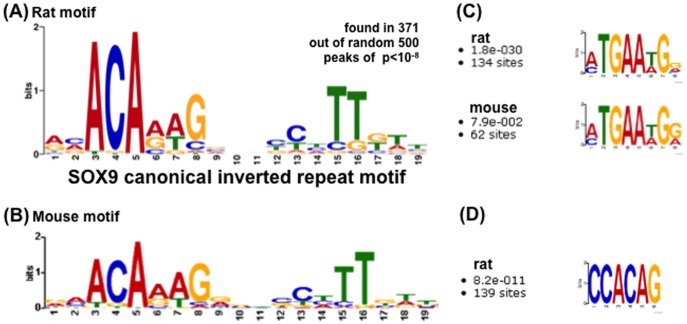
Consensus binding motif of SOX9-interacting sites detected in chondrocytes. A) The MEME program was used to search for a common motif among 500 random peaks identified by ChIP-Seq experiments (p value <10^−8^) in RCS cells. The motif was found within 100 bp on either side of the top of the peak in 371 of the genomic SOX9-interaction sites. The motif is an inverted repeat of a canonical motif of six bases on each side with an interval of four bases. B) A search for the same motif within 100 bp on either side of the top of 800 of the strongest binding peaks (p value ≤1e-5, fold ≤2) identified the motif in rib chondrocytes. C) A secondary motif was found in the same binding peaks shown in B). D) In RCS cells, a third motif was found to be similar to a RUNX1 consensus binding site.

SOX9 is thought to regulate the basal transcriptional machinery by recognizing and interacting with sites that can be far from the transcription start site. To examine whether a common binding motif was detected in the ChIP-Seq peaks, we analyzed the 500 peaks that had the lowest p value by the MEME motif search program. In RCS cells, a consensus inverted motif ACAAAG/CTTTGT with four bases between them was identified within 100 bp on either side of the top of the peak in 371 of the 500 peaks (74%) (Figure 6A). This motif corresponds to an inverted repeat that has previously been shown to exist in SOX9-binding sites of several cartilage ECM genes, including *Acan*, *Col11a2*, *Col9a1*, and *Matrilin-3*, by *in vitro* binding assays [31], [35]–[39]. When we searched by MEME for motifs within 100 bp on either side of the top of the peak for the 800 strongest binding peaks (p value ≤10^−5^, fold ≤2) in rib chondrocytes, we found motif similar to that shown above in 242 peaks (Figure 6B). This indicates that SOX9 mainly interacts as a homodimer with an inverted DNA repeat motif in the genome of chondrocytes.

Since SOX9 could regulate the activity of target genes by participating in protein complexes, other common motifs might have been expected to be detected near SOX9-binding sites. A search for such conserved motifs in the ChIP-Seq peaks revealed the existence of a RUNX1 motif near SOX-binding sites in 139 of 800 peaks in RCS cells (Figure 6D). In related to this result, the regulation of CBFb-RUNX1 transcriptional program has been shown to be involved in chondrocyte differentiation [34]. In addition, a (A/C)TGAA(T/A)G(G/A) motif was identified by the same search program in 62 and 134 peaks, respectively, of the 800 strongest ChIP-Seq peaks within 100 bp on either side of the top of the peak in rib chondrocytes and RCS cells (Figure 6C), but this motif has not yet been identified as a consensus binding site for a known transcription factor by using JASPAR (http://jaspar.genereg.net), the largest open-access database describing the binding preference of transcription factors from multiple species.

### Possible direct targets of SOX9 suggested by RNA-Seq and Chip-Seq results

We next examined which genes identified as differentially regulated by SOX9 in RNA-Seq experiments are possible direct targets of SOX9. We determined the percentage of these genes in each of the functional categories that have SOX9-interaction sites. Note that, in this analysis, only the interaction sites that fell between 50 Kb 5′ and 50 Kb 3′ of the genes were considered. In the genes whose expression was decreased by more than 6-fold by the removal of SOX9, the overall ratios of genes that have interaction sites are 47% in mouse chondrocytes and 43% in RCS cells (Table 1). Fifty percent of the genes in the ECM category showed peaks in mouse chondrocytes and 56% in RCS; among the transferase genes, 89% and 67% showed peaks, and in the transporter group the percentages were 25% and 38% (Figure 2). Thus roughly half of the genes regulated by SOX9 in chondrocytes are likely to be direct targets of SOX9. They include not only the genes encoding ECM proteins, which were expected, but also other genes encoding transporters, transferases, receptors, and others.

Of the genes with SOX9-interaction peaks in RCS cells and those with peaks in mouse rib chondrocytes, 24% of rat genes and 27% of mouse genes are common between the two species (Figure S3). There are probably several reasons for the less-than-complete overlap of SOX9-interaction sites between the two cell types. RCS cells are cancer cells, which proliferate fast with a doubling time of around 18 hours. On the other hand, primary rib chondrocyte cells consist of a mixed population of proliferating, prehypertrophic, and hypertrophic chondrocytes. Recent reports have shown that SOX9 regulates programing of pancreatic and liver progenitors [40], [41], and mutations in SOX9 have been identified in colorectal cancer cells [42]. In cancer cells, SOX9 might regulate genes associated with tumorigenesis [43]. The functional differences between the two cell types suggest that not only common genes but also genes unique to either cell type could be activated by SOX9. Finally, it is at least possible that some direct targets of SOX9 contain interaction sites at distances more than 50 Kb 5′ or 3′ from the gene in one species but not in the other. The finding that many non-common genes in the two cell types contain SOX9 interaction peaks could be attributed to the likely differences in overall gene expression profiles of these two cell types and to the existence of genes that are active in only one cell type not in the other. However, the consensus motif detected in the peaks of rib chondrocytes is very similar to that detected in RCS cells, namely a canonical SOX9-binding inverted repeat with the same interval between the repeats. Hence the above differences are likely not due to differences in the interaction motifs in the two cell types. We also noted that the proportion of genes that have SOX9-interaction sites in each of the gene categories identified by the RNA-Seq results (Table 1) were very similar in the two cell types.

Several SOX9-interacting proteins have been identified, including p300, Tip60, Arid5a, Znf219, and Med25 [44]–[48]. These proteins may participate in protein complexes that regulate SOX9-activated gene expression.

Even though there is no evidence that SOX5 or SOX6 interacts directly with SOX9, there is strong genetic and biochemical evidence that they collaborate with SOX9 at specific steps in chondrocyte differentiation. It is possible that SOX5 and SOX6 bind near SOX9-binding sites and regulate gene expression together with SOX9 [49]. Their DNA-binding sequence motifs are similar to those of SOX9 and so far cannot be distinguished from the SOX9-interaction sites. The nature of the contributions of SOX5 and SOX6 to the composition of the SOX9-protein complex and their function in transcriptional activation need to be further clarified.

One more important issue related to the SOX9-binding sites has to do with the kind of chromatin structures that exist at these sites and whether these have common features. It has been shown previously that the SOX9-interaction sites in intron 6 of *Col2a1* are depleted of nucleosomes, as suggested by the absence of histone H3 at these sites [31]. It might be speculated that the chromatin status of SOX9-interaction sites might be either nucleosome depleted or enriched in trimethylated H3K4 or H3K36, similar to actively transcribed chromatin. Nucleosome depletion or enrichment in trimethylated H3K4 or K36 could allow easy access by SOX9. The status of chromatin at SOX9-binding sites throughout the genome obviously remains to be clarified.

SOX9 is expressed in nonchondrogenic tissues such as gonads, embryonic pancreas, hair follicles, prostate, and paneth cells of the intestine. Several genes have been identified as direct targets of SOX9 in these tissues [50]–[53], and these targets in other cell types do not have the SOX9-interaction sites we identified in chondrocytes. The present study has presented evidence that SOX9 regulates a specific set of genes in chondrocytes and controls the differentiation of these cells by activating not only cartilage ECM genes but also genes encoding ECM modification enzymes, membrane receptors, transporters, and other related proteins. A large proportion of these genes have been shown to harbor characteristic inverted repeats to which SOX9 binds as a homodimer to perform its transcriptional role as a master regulatory factor of chondrocyte differentiation.

## Materials and Methods

### Ethics statement

All experiments in this study carried out in strict accordance with the recommendation in the Guide for the Care and Use of Laboratory Animals of the National Institute of Health, USA. The procedures were approved by the Institutional Animal Care and Use Committee of The University of Texas M.D. Anderson Cancer Center (IACUC No. 1088–7638).

### Cell culture and adenovirus infection

RCS cells [32] and HEK293T cells were cultured at 37°C in Dulbecco modified Eagle medium supplemented with 10% fetal bovine serum. Rib chondrocytes were isolated from 4-day-old *Sox9^flox/flox^* mice, in which the DNA segment containing exons 2 and 3 of the *Sox9* gene is flanked by *loxP* sites (i.e., floxed) [2] or wild-type mice and were cultured as described previously [54]. After 1 day of culture, the semi-confluent cells were infected with Ad-CMV-*Cre* or Ad-CMV-Null virus. Forty-eight hours after infection, the cells were harvested and the expression of *Sox9* in total RNA was determined by reverse-transcription quantitative polymerase chain reaction (RT-qPCR). The titers of the adenoviruses, prepared by the Genetically Engineered Mouse Core facility at Baylor College of Medicine, were obtained by using an Adeno-X rapid titer assay kit (Clontech, Mountain View, CA) according to the manufacturer's protocol.

### Measurement of mRNA expression

To measure the mRNA expression level, total RNA was extracted from primary chondrocytes using Trizol reagent (Invitrogen, Carlsbad, CA) according to the manufacturer's protocol. cDNA was prepared from the mRNA using AMV reverse transcriptase (Invitrogen). qPCR was performed using primers specific for each RNA, SYBR Master Mix, and an ABI 7900 real-time PCR system (Applied Biosystems, Foster City, CA), as described elsewhere [31]. The difference in Ct values (delta Ct) among the Ct values of each sample was calculated.

### RNA-Seq analysis

The total RNA was purified by use of Trizol, and the polyA-containing mRNA was prepared by using oligo dT resin and then transcribed to cDNA using a cDNA preparation kit according to the manufacturer's protocol. After the adaptors were ligated to the cDNA, the cDNA library was prepared by PCR according to the manufacturer's protocol. The second-generation sequencing was done from both ends by a Hi-Seq-2000 sequencer using the Illumina platform in the DNA Analysis Core facility at The University of Texas M.D. Anderson Cancer Center. The sequences obtained were aligned to the mouse genome sequence.

### ChIP-Seq and ChIP-qPCR analysis

ChIP was performed according to the previously described method [31] using a ChIP assay kit (Millipore, Billerica, MA). Briefly, cultured mouse rib chondrocytes or RCS cells were fixed with formaldehyde, and the chromatin prepared by sonication was treated with a rabbit anti-SOX9 antibody (Millipore, AB5809) or nonspecific rabbit IgG antibody. Before making the libraries, the qualities of the resultant DNA fragments were verified by use of ChIP-qPCR using the SYBR Green PCR Master Mix and an ABI 7900HT qPCR system using ChIP-DNA as a template. The primers used for this purpose were as follows: m*Col2a1* intron 1, TGAGGCTTGTTTGCGTTGAG and AGGGCATGGTGACTCAGATG; m*Col2a1* intron 7 CCCGTCGTGCGGTTAATT and ACTGCTCTTCCAGAGAAACACAAGT and corresponded to two previously characterized SOX9-interaction sites in the *Col2a1* gene. The libraries of ChIP-DNA were made according to the manufacturer's protocol and second-generation sequencing was performed as already described. The sequences obtained were aligned with the mouse or rat genome sequences. The data were analyzed using input DNA pattern as a control, and ChIP-Seq data from both cell types were annotated by mouse genes, because many rat genes have not been annotated yet.

### Plasmid construction and reporter assay

*Sox9* fragments containing 1 kb of its promoter and exon1 and 750 bp surrounding the SOX9-interacting site at −80 Kb were prepared by PCR using a BAC clone from the BACPAC Resource Center (Oakland, CA) as a template. The internal ribosome entry site was inserted in the multi cloning sites of the pGL3 vector (Promega, Madison, WI), and the two fragments were inserted into the 5′ region of that site. In the control plasmid, only the fragment comprising promoter and exon1 fragment was inserted. The sequences of all constructs were verified by DNA sequencing. The reporter assay was done as described previously [31]. Briefly, 1.5×10^5^ 293T cells were inoculated into each well of a 12-well plate. After 24 hours of incubation, the cells were cotransfected with the reporter plasmid construct (170 ng/well), a SOX9-expressing plasmid (300 ng/well), and a CMV-*renilla* luciferase plasmid (2 ng/well), which was used as a transfection control, using Fugene 6 (Clontech). After a 36-hour incubation, the luciferase activity was measured using a dual luciferase assay system (Promega). Each value in the reporter assay is presented as the fold-increase in *firefly* luciferase activity per *renilla* luciferase activity units from three independent experiments.

## Supporting Information

Figure S1 **Classification of genes whose expression was increased after removal of Sox9.** Genes whose expression was increased by more than 4-, or 8-fold by the removal of SOX9 were classified into functional categories. The experiments and analysis were done as shown in Fig. 2.
(TIFF)

Figure S2 **The sequence alignment of −200 Kb peak of RCS cells and −250 Kb peak of mouse rib chondrocytes.** The 601 base pairs surrounding −200 Kb and −250 Kb peaks of mouse rib chondrocytes and RCS cells, respectively, were aligned.
(TIFF)

Figure S3 **The number of genes that have SOX9-interaction sites in RCS cells and rib chondrocytes.**
(TIFF)

Table S1 **Classification of genes with decreased expression after removal of SOX9.**
(DOC)

Table S2 **Classification of genes with increased expression after removal of Sox9.**
(DOC)

Table S3 **Genes with decreased expression by more than 4-fold after removal of SOX9, classified by the David classification program.** This table also shows the genes for which there is some evidence of association with human disease according to the University of Copenhagen Disease Data Base (http//www.disease.jensenlab.org).
(DOC)

Table S4 **Genes with increased expression by more than 8-fold after removal of** ***Sox9.***
(DOC)
