# Correction: SOX9 Regulates Multiple Genes in Chondrocytes, Including Genes Encoding ECM Proteins, ECM Modification Enzymes, Receptors, and Transporters

**DOI:** 10.1371/journal.pone.0143156

**Published:** 2015-11-12

**Authors:** Chun-do Oh, Yue Lu, Shoudan Liang, Yuko Mori-Akiyama, Di Chen, Benoit de Crombrugghe, Hideyo Yasuda

The Data Availability statement is incorrect. The correct statement is: All relevant data are available via NCBI's Gene Expression Omnibus (GEO) (accession number GSE73984).
