# Supplementary figures and images for: SOX9 Regulates Multiple Genes in Chondrocytes, Including Genes Encoding ECM Proteins, ECM Modification Enzymes, Receptors, and Transporters
Source: PLoS One. 2014 Sep 17;9(9):e107577. doi: 10.1371/journal.pone.0107577 (PMC4168005; doi:10.1371/journal.pone.0107577)

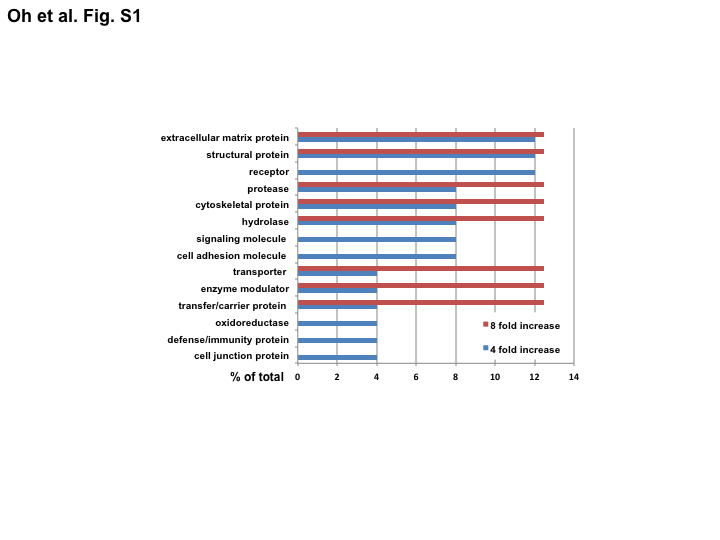

Supplement: Figure S1 — Classification of genes whose expression was increased after removal of Sox9. Genes whose expression was increased by more than 4-, or 8-fold by the removal of SOX9 were classified into functional categories. The experiments and analysis were done as shown in Fig. 2. (TIFF) [file pone.0107577.s001.tiff]

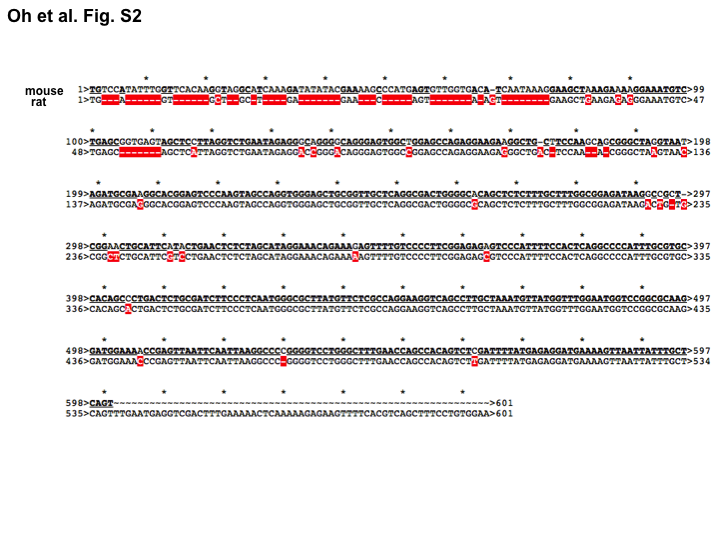

Supplement: Figure S2 — The sequence alignment of −200 Kb peak of RCS cells and −250 Kb peak of mouse rib chondrocytes. The 601 base pairs surrounding −200 Kb and −250 Kb peaks of mouse rib chondrocytes and RCS cells, respectively, were aligned. (TIFF) [file pone.0107577.s002.tiff]

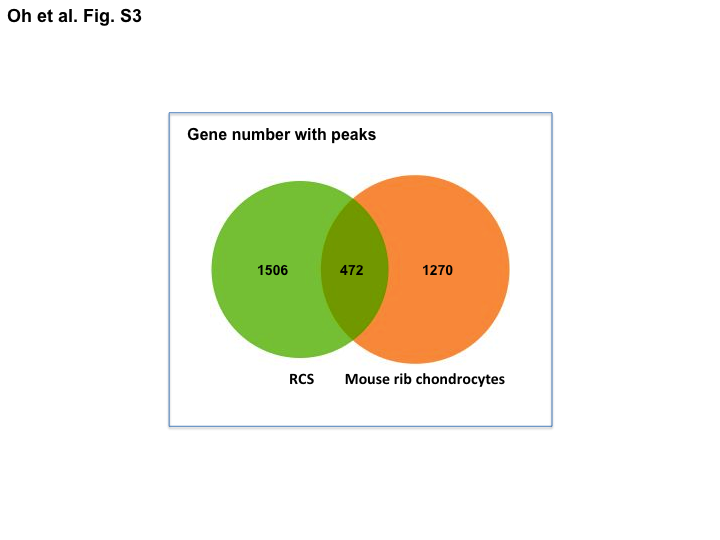

Supplement: Figure S3 — The number of genes that have SOX9-interaction sites in RCS cells and rib chondrocytes. (TIFF) [file pone.0107577.s003.tiff]
