# Supplementary material for: SOX9 Regulates Multiple Genes in Chondrocytes, Including Genes Encoding ECM Proteins, ECM Modification Enzymes, Receptors, and Transporters
Source: PLoS One. 2014 Sep 17;9(9):e107577. doi: 10.1371/journal.pone.0107577 (PMC4168005; doi:10.1371/journal.pone.0107577)
Supplement: Table S1 — Classification of genes with decreased expression after removal of SOX9. (DOC) [file pone.0107577.s004.doc]

**Table S1. Classification of genes with decreased expression after removal of SOX9**

| **categories** | 4Xdecrease | % of Total | 6Xdecrease | % of Total | 8Xdecrease | % of Total |
| --- | --- | --- | --- | --- | --- | --- |
|  | # of genes |  | # of genes |  | # of genes |  |
| extracellular matrix protein | 29 | 10 | 19 | 14 | 14 | 18 |
| receptor | 35 | 12 | 18 | 13 | 11 | 14 |
| transporter | 26 | 9 | 14 | 10 | 8 | 10 |
| cell adhesion molecule | 16 | 5 | 9 | 7 | 8 | 10 |
| transferase | 25 | 8 | 9 | 7 | 6 | 8 |
| defense/immunity protein | 14 | 5 | 10 | 7 | 6 | 8 |
| surfactant | 7 | 2 | 6 | 4 | 6 | 8 |
| signaling molecule | 17 | 6 | 8 | 6 | 4 | 5 |
| kinase | 11 | 4 | 4 | 3 | 3 | 4 |
| oxidoreductase | 15 | 5 | 6 | 4 | 2 | 3 |
| enzyme modulator | 11 | 4 | 4 | 3 | 2 | 3 |
| transcription factor | 16 | 5 | 5 | 4 | 2 | 3 |
| protease | 9 | 3 | 4 | 3 | 1 | 1 |
| cytoskeletal protein | 7 | 2 | 3 | 2 | 1 | 1 |
| ligase | 7 | 2 | 1 | 1 | 1 | 1 |
| nucleic acid binding | 11 | 4 | 3 | 2 | 1 | 1 |
| calcium-binding protein | 9 | 3 | 4 | 3 | 1 | 1 |
| hydrolase | 17 | 6 | 5 | 4 | 1 | 1 |
| transfer/carrier protein | 9 | 3 | 2 | 1 | 1 | 1 |
| structural protein | 2 | 1 | 2 | 1 | 1 | 1 |
| transmembrane receptor regulatory/adaptor protein | 2 | 1 | 1 | 1 |  | 0 |
| lyase | 2 | 1 |  | 0 |  | 0 |
| membrane traffic protein | 1 | 0 |  | 0 |  | 0 |
| phosphatase | 3 | 1 | 1 | 1 |  | 0 |
| cell junction protein | 1 | 0 |  | 0 |  | 0 |
| isomerase | 1 | 0 |  | 0 |  | 0 |
| Total number | 303 | 100 | 138 | 100 | 80 | 100 |
