# Supplementary material for: SOX9 Regulates Multiple Genes in Chondrocytes, Including Genes Encoding ECM Proteins, ECM Modification Enzymes, Receptors, and Transporters
Source: PLoS One. 2014 Sep 17;9(9):e107577. doi: 10.1371/journal.pone.0107577 (PMC4168005; doi:10.1371/journal.pone.0107577)
Supplement: Table S2 — Classification of genes with increased expression after removal of Sox9. (DOC) [file pone.0107577.s005.doc]

**Table S2. Classification of genes with increased expression after removal of Sox9**

| | **categories** | 4xincreased | **% of Total** | 8xincreased | **% of Total** | | --- | --- | --- | --- | --- | | # of genes | # of genes | | extracellular matrix protein | 3 | 12 | 1 | 12.5 | | structural protein | 3 | 12 | 1 | 12.5 | | receptor | 3 | 12 | 0 | 0 | | protease | 2 | 8 | 1 | 12.5 | | cytoskeletal protein | 2 | 8 | 1 | 12.5 | | hydrolase | 2 | 8 | 1 | 12.5 | | signaling molecule | 2 | 8 | 0 | 0 | | cell adhesion molecule | 2 | 8 | 0 | 0 | | transporter | 1 | 4 | 1 | 12.5 | | enzyme modulator | 1 | 4 | 1 | 12.5 | | transfer/carrier protein | 1 | 4 | 1 | 12.5 | | oxidoreductase | 1 | 4 | 0 | 0 | | defense/immunity protein | 1 | 4 | 0 | 0 | | cell junction protein | 1 | 4 | 0 | 0 | | **Total number** | **25** | **100%%** | **8** | **100** | |
| --- | --- | --- | --- | --- | --- | --- | --- | --- | --- | --- | --- | --- | --- | --- | --- | --- | --- | --- | --- | --- | --- | --- | --- | --- | --- | --- | --- | --- | --- | --- | --- | --- | --- | --- | --- | --- | --- | --- | --- | --- | --- | --- | --- | --- | --- | --- | --- | --- | --- | --- | --- | --- | --- | --- | --- | --- | --- | --- | --- | --- | --- | --- | --- | --- | --- | --- | --- | --- | --- | --- | --- | --- | --- | --- | --- | --- | --- | --- | --- | --- | --- | --- |
