# Supplementary material for: SOX9 Regulates Multiple Genes in Chondrocytes, Including Genes Encoding ECM Proteins, ECM Modification Enzymes, Receptors, and Transporters
Source: PLoS One. 2014 Sep 17;9(9):e107577. doi: 10.1371/journal.pone.0107577 (PMC4168005; doi:10.1371/journal.pone.0107577)
Supplement: Table S3 — Genes with decreased expression by more than 4-fold after removal of SOX9, classified by the David classification program. This table also shows the genes for which there is some evidence of association with human disease according to the University of Copenhagen Disease Data Base (http//www.disease.jensenlab.org). (DOC) [file pone.0107577.s006.doc]

**Table S**3. Genes with decreased expression by more than 4-fold after removal of SOX9, classified by the David classification program

| | **ECM proteins** |  |  |  |  | | --- | --- | --- | --- | --- | |  |  | **Humanskeletal disease** | **SOX9 binding mouse** | **SOX9 binding rat** | | **Col2a1** | **Collagen alpha-1(ll) chain;** | ***** | ***** | ***** | | **Col9a1** | **Collagen alpha-1(IX) chain;Col9a1;ortholog** | ***** | ***** | ***** | | **Col11a1** | **Collagen alpha-1(XI) chain;Col11a1;ortholog** | ***** | ***** | ***** | | **Col27a1** | **Collagen alpha-1(XXVII) chain;Col27a1;ortholog** | ***** | ***** | ***** | | **Col11a2** | **Collagen alpha-2(XI) chain;Col11a2;ortholog** | ***** |  |  | | **Col9a2** | **Collagen alpha-2(IX) chain;Col9a2;ortholog** | ***** | ***** | ***** | | **Col9a3** | **collagen, type IX, alpha 3** | ***** | ***** | ***** | | **Col10a1** | **Collagen alpha-1(X) chain;Col10a1;ortholog** | ***** |  |  | | **Acan** | **Aggrecan core protein;Acan;ortholog** | ***** | ***** | ***** | | **Matn3** | **Matrilin-3;Matn3;ortholog** | ***** | ***** | ***** | | **Matn4** | **Matrilin-4;Matn4;ortholog** | ***** |  | ***** | | **Ucma** | **Unique cartilage matrix-associated protein C-terminal fragment;Ucma;ortholog** | ***** | ***** |  | | **Hapln1** | **Hyaluronan and proteoglycan link protein 1;Hapln1;ortholog** | ***** |  | ***** | | **Dcn** | **Neuronal migration protein doublecortin;Dcx;ortholog** | ***** |  |  | | **Prelp** | **Prolargin;Prelp;ortholog** | ***** | ***** |  | | **Nid2** | **Nidogen-2;Nid2;ortholog** |  |  | ***** | | **Lgals3** | **Galectin-3;Lgals3;ortholog** |  | ***** | ***** | | **Eln** | **Elastin;Eln;ortholog** | ***** |  |  | | **Mgp** | **Matrix Gla protein;Mgp;ortholog** |  | ***** | ***** | | **Lrrc32** | **leucine rich repeat containing 32** |  |  |  | |  |  |  |  |  | | **Secreted proteins** |  |  |  |  | | **Grb14** | **Growth factor receptor-bound protein 14;Grb14;ortholog** |  |  |  | | **Fibin** | **Fin bud initiation factor homolog;Fibin;ortholog** |  |  |  | | **Gdf10** | **Bone morphogenetic protein 3b;Gdf10;ortholog** |  |  |  | | **Bmp6** | **Bone morphogenetic protein 6;Bmp6;ortholog** | ***** | ***** |  | | **Nog** | **Noggin;Nog;ortholog** | ***** |  |  | | **C1qtnf3** | **Complement C1q tumor necrosis factor-related protein 3;C1qtnf3;ortholog** | ***** |  |  | | **Trf** | **Interleukin-5;Il5;ortholog** |  |  |  | | **Lcn2** | **Neutrophil gelatinase-associated lipocalin;Lcn2;ortholog** |  | ***** |  | | **Crispld1** | **Cysteine-rich secretory protein LCCL domain-containing 1;Crispld1;ortholog** |  |  |  | | **Cp** | **Ceruloplasmin;Cp;ortholog** |  | ***** |  | | **Mia1** | **Melanoma-derived growth regulatory protein;Mia;ortholog** | ***** | ***** | ***** | | **Metrn** | **Meteorin;Metrn;ortholog** |  |  |  | | **Lect1** | **Chondrosurfactant protein;Lect1;ortholog** | ***** | ***** | ***** | | **Slpi** | **Antileukoproteinase;Slpi;ortholog** |  |  | ***** | | **Serpina3n** | **Serine protease inhibitor A3N;Serpina3n;ortholog** |  |  |  | | **Scube1** | **Signal peptide, CUB and EGF-like domain-containing protein 1;Scube1;ortholog** |  |  |  | | **Stc2** | **Stanniocalcin-2;Stc2;ortholog** |  |  |  | |  |  |  |  |  | | **Penk** | **Met-enkephalin-Arg-Phe;Penk;ortholog** |  |  |  | | **Frzb** | **Secreted frizzled-related protein 3;Frzb;ortholog** | ***** |  | ***** | | **Arsi** | **Arylsulfatase I;Arsi;ortholog** |  | ***** | ***** | | **Chi3l1** | **Chitinase-3-like protein 1;Chi3l1;ortholog** | ***** |  |  | | **Enpp2** | **Ectonucleotide pyrophosphatase/phosphodiesterase family member 2;Enpp2;ortholog** | ***** | ***** |  | | **Pla2g12a** | **Group XIIA secretory phospholipase A2;Pla2g12a;ortholog** |  |  |  | | **Loxl4** | **Lysyl oxidase homolog 4;Loxl4;ortholog** |  | ***** |  | | **Maged2** | **similar to melanoma antigen family D, 2; melanoma antigen, family D, 2** |  |  |  | |  |  |  |  |  | | **Intrinsic membrane proteins** |  |  |  |  | | **ECM modification enzymes** |  |  |  |  | | **Leprel1** | **Prolyl 3-hydroxylase 2;Leprel1;ortholog** | ***** | ***** |  | | **Glt25d2** | **Glycosyltransferase 25 family member 2;Glt25d2;ortholog** |  | ***** | ***** | | **Gfpt1** | **Glucosamine--fructose-6-phosphate aminotransferase [isomerizing] 1;Gfpt1;ortholog** | ***** | ***** | ***** | | **Moxd1** | **DBH-like monooxygenase protein 1;Moxd1;ortholog** |  |  |  | | **Extl1** | **Exostosin-like 1;Extl1;ortholog** | ***** | ***** | ***** | | **B4galnt3** | **N-acetyl-beta-glucosaminyl-glycoprotein 4-beta-N-acetylgalactosaminyltransferase 2;B4galnt3;ortholog** |  | ***** | ***** | | **Chst11** | **Carbohydrate sulfotransferase 11;Chst11;ortholog** | ***** | ***** | ***** | | **Mgat4a** | **Alpha-1,3-mannosyl-glycoprotein 4-beta-N-acetylglucosaminyltransferase A soluble form;Mgat4a;ortholog** |  | ***** | ***** | | **Smpd3** | **Sphingomyelin phosphodiesterase 3;Smpd3;ortholog** | ***** | ***** | ***** | | **Xylt1** | **Xylosyltransferase 1;Xylt1;ortholog** | ***** | ***** | ***** | | **B3galnt2** | **UDP-GalNAc:beta-1,3-N-acetylgalactosaminyltransferase 2;B3galnt2;ortholog** |  |  |  | | **Pcolce2** | **Procollagen C-endopeptidase enhancer 2;Pcolce2;ortholog** | ***** | ***** |  | | **Membrane receptors** |  |  |  |  | | **Steap3** | **Metalloreductase STEAP3;Steap3;ortholog** |  |  |  | | **Fgfr3** | **Fibroblast growth factor receptor 3;Fgfr3;ortholog** | ***** | ***** |  | | **Fgfrl1** | **Fibroblast growth factor receptor-like 1;Fgfrl1;ortholog** | ***** | ***** |  | | **Fzd9** | **Frizzled-9;Fzd9;ortholog** | ***** | ***** | ***** | | **Itga10** | **integrin, alpha 10** | ***** |  |  | | **Jph1** | **Junctophilin-1;Jph1;ortholog** |  |  |  | | **Sdk2** | **Protein sidekick-2;Sdk2;ortholog** |  | ***** | ***** | | **Crlf3** | **Cytokine receptor-like factor 3;Crlf3;ortholog** |  |  |  | | **Transporter, Channel** |  |  |  |  | | **Trpv4** | **Transient receptor potential cation channel subfamily V member 4;Trpv4;ortholog** | ***** | ***** | ***** | | **Kcns1** | **Potassium voltage-gated channel subfamily S member 1;Kcns1;ortholog** |  | ***** | ***** | | **Kctd15** | **BTB/POZ domain-containing protein KCTD15;Kctd15;ortholog** |  |  |  | | **Kcna6** | **Potassium voltage-gated channel subfamily A member 6;Kcna6;ortholog** |  |  | ***** | | **Tmem20** | **Transmembrane protein 20;Tmem20;ortholog** |  |  |  | | **Slc6a9** | **Sodium- and chloride-dependent glycine transporter 1;Slc6a9;ortholog** |  |  |  | | **Slc16a2** | **Monocarboxylate transporter 8;Slc16a2;ortholog** |  |  |  | | **Slc25a39** | **Solute carrier family 25 member 39;Slc25a39;ortholog** |  |  |  | | **Slc39a14** | **Zinc transporter ZIP14;Slc39a14;ortholog** |  | ***** | ***** | | **Slc7a2** | **Low affinity cationic amino acid transporter 2;Slc7a2;ortholog** |  |  | ***** | | **Slc1a5** | **Neutral amino acid transporter B(0);Slc1a5;ortholog** |  | ***** |  | | **Slc7a5** | **Large neutral amino acids transporter small subunit 1;Slc7a5;ortholog** |  |  |  | | **Slc26a2** | **Sulfate transporter;Slc26a2;ortholog** | ***** | ***** | ***** | | **Slc38a3** | **Sodium-coupled neutral amino acid transporter 3;Slc38a3;ortholog** |  | ***** | ***** | | **Atp1a1** | **Sodium/potassium-transporting ATPase subunit alpha-1;Atp1a1;ortholog** |  |  | ***** | | **Chac1** | **Cation transport regulator-like protein 1;Chac1;ortholog** |  |  |  | | **Others** |  |  |  |  | | **Grb2** | **Growth factor receptor-bound protein 2;Grb2;ortholog** |  | ***** |  | | **Bambi** | **BMP and activin membrane-bound inhibitor homolog;Bambi;ortholog** |  |  |  | | **Erlin1** | **Erlin-1;Erlin1;ortholog** |  |  |  | | **Gpr125** | **Probable G-protein coupled receptor 125;Gpr125;ortholog** |  |  | ***** | | **Gpr64** | **G-protein coupled receptor 64;Gpr64;ortholog** |  |  |  | | **Npc1** | **Niemann-Pick C1 protein;Npc1;ortholog** |  |  |  | | **3110079O15Rik** | **RIKEN cDNA 3110079O15 gene,C2orf82,single pass memmbrane protein** |  | ***** | ***** | | **A330049M08Rik** | **RIKEN cDNA A330049M08 gene** |  | ***** |  | | **D630045J12Rik** | **RIKEN cDNA D630045J12 gene** |  | ***** | ***** | | **Avpr1a** | **Vasopressin V1a receptor;Avpr1a;ortholog** |  |  |  | | **Cmklr1** | **Chemokine receptor-like 1;Cmklr1;ortholog** |  | ***** | ***** | | **Ccdc109a** | **Coiled-coil domain-containing protein 109A;Ccdc109a;ortholog** |  |  |  | | **AU040320** | **expressed sequence AU040320** |  |  |  | | **Herpud1** | **Homocysteine-responsive endoplasmic reticulum-resident ubiquitin-like domain member 1 protein;Herpud1** |  |  |  | | **Ldlrad3** | **Low-density lipoprotein receptor class A domain-containing protein 3;Ldlrad3;ortholog** |  | ***** | ***** | | **Lrp4** | **Low-density lipoprotein receptor-related protein 4;Lrp4;ortholog** | ***** |  |  | | **Mest** | **Mesoderm-specific transcript protein;Mest;ortholog** | ***** |  |  | | **Plxnb1** | **Plexin-B1;Plxnb1;ortholog** |  | ***** | ***** | | **Rhbdd1** | **Rhomboid domain-containing protein 1;Rhbdd1;ortholog** |  | ***** |  | | **Scara5** | **Scavenger receptor class A member 5;Scara5;ortholog** |  |  | ***** | | **Sema6a** | **Semaphorin-6A;Sema6a;ortholog** |  |  | ***** | | **Serinc5** | **Serine incorporator 5;Serinc5;ortholog** |  |  |  | | **Nrxn2** | **neurexin II** |  |  | ***** | | **Sept5** | **Septin-5;Sept5;ortholog** | ***** |  |  | |  |  |  |  |  | | **Aminotransferase** |  |  |  |  | | **Bcat1** | **Branched-chain-amino-acid aminotransferase, cytosolic;Bcat1;ortholog** |  | ***** |  | | **Oat** | **Ornithine aminotransferase, mitochondrial;Oat;ortholog** |  | ***** | ***** | | **Gpt2** | **Alanine aminotransferase 2;Gpt2;ortholog** |  |  | ***** | | **Got1** | **Aspartate aminotransferase, cytoplasmic;Got1;ortholog** |  | ***** |  | |  |  |  |  |  | | **Folic acid metabolism** |  |  |  |  | | **Aldh1l2** | **Probable 10-formyltetrahydrofolate dehydrogenase ALDH1L2;Aldh1l2;ortholog** |  | ***** |  | | **Mthfd2** | **Methenyltetrahydrofolate cyclohydrolase;Mthfd2;ortholog** |  |  |  | | **Mthfd1l** | **Monofunctional C1-tetrahydrofolate synthase, mitochondrial;Mthfd1l;ortholog** |  |  | ***** | |  |  |  |  |  | | **Mitochondria envelope** |  |  |  |  | | **Gls** | **Glutaminase liver isoform, mitochondrial;Gls2;ortholog** |  | ***** | ***** | | **Prdx5** | **Peroxiredoxin-5, mitochondrial;Prdx5;ortholog** |  | ***** |  | | **Pck2** | **Phosphoenolpyruvate carboxykinase [GTP], mitochondrial;Pck2;ortholog** |  | ***** |  | | **Prdx6** | **Peroxiredoxin-6;Prdx6;ortholog** |  |  |  | | **Ndufa10** | **NADH dehydrogenase [ubiquinone] 1 alpha subcomplex subunit 10, mitochondrial;Ndufa10;ortholog** |  |  |  | | **Ndufa2** | **NADH dehydrogenase [ubiquinone] 1 alpha subcomplex subunit 2;Ndufa2;ortholog** |  | ***** |  | | **Cox17** | **Cytochrome c oxidase copper chaperone;Cox17;ortholog** |  | ***** |  | | **Cyp26b1** | **cytochrome P450, family 26, subfamily b, polypeptide 1** | ***** |  |  | |  |  |  |  |  | | **Calcium binding protein** |  |  |  |  | | **Efha1** | **EF-hand domain-containing family member A1;Efha1;ortholog** |  |  |  | | **Ehd3** | **EH domain-containing protein 3;Ehd3;ortholog** |  |  | ***** | | **Rab11fip4** | **Rab11 family-interacting protein 4;Rab11fip4;ortholog** |  | ***** | ***** | |  |  |  |  |  | | **Actin filament regulator** |  |  |  |  | | **Avil** | **Advillin;Avil;ortholog** |  |  |  | | **Scin** | **Adseverin;Scin;ortholog** |  | ***** |  | |  |  |  |  |  | | **Transcription factor regulator** | |  |  |  | | **Alx1** | **ALX homeobox protein 1;Alx1;ortholog** | ***** | ***** | ***** | | **Cebpd** | **CCAAT/enhancer-binding protein delta;Cebpd;ortholog** |  |  |  | | **Sox6** | **Transcription factor SOX-6;Sox6;ortholog** | ***** | ***** | ***** | | **Sox9** | **Transcription factor SOX-9;Sox9;ortholog** | ***** |  |  | | **Atf4** | **Cyclic AMP-dependent transcription factor ATF-4;Atf4;ortholog** | ***** | ***** |  | | **Foxd1** | **Forkhead box protein D1;Foxd1;ortholog** |  | ***** |  | | **Ing2** | **Inhibitor of growth protein 2;Ing2;ortholog** |  |  |  | | **Nr4a1** | **Nuclear receptor subfamily 4 group A member 1;Nr4a1;ortholog** |  |  |  | | **Plagl1** | **pleiomorphic adenoma gene-like 1** |  |  |  | | **Nfx1** | **Transcriptional repressor NF-X1;Nfx1;ortholog** |  |  | ***** | |  |  |  |  |  | | **Zinc binding protein** |  |  |  |  | | **Zim1** | **zinc finger, imprinted 1** |  |  |  | | **Mfi2** | **Melanotransferrin;Mfi2;ortholog** |  |  | ***** | | **Zfp385c** | **Zinc finger protein 385C;Znf385c;ortholog** |  | ***** |  | | **Zfp385b** | **Zinc finger protein 385B;Znf385b;ortholog** |  | ***** |  | | **Zcchc5** | **zinc finger, CCHC domain containing 5** |  |  | ***** | |  |  |  |  |  | | **ATP binding proteins** |  |  |  |  | | **Papss2** | **Adenylyl-sulfate kinase;Papss2;ortholog** | ***** | ***** | ***** | | **Ulk2** | **Serine/threonine-protein kinase ULK2;Ulk2;ortholog** |  |  |  | | **Wnk4** | **Serine/threonine-protein kinase WNK4;Wnk4;ortholog** |  |  |  | | **Pkn3** | **Serine/threonine-protein kinase N3;Pkn3;ortholog** |  |  |  | | **Stk39** | **STE20/SPS1-related proline-alanine-rich protein kinase;Stk39;ortholog** | ***** | ***** |  | | **Nme2** | **Nucleoside diphosphate kinase B;Nme2;ortholog** |  |  |  | | **Cmpk1** | **UMP-CMP kinase;Cmpk1;ortholog** |  |  |  | | **Asns** | **Asparagine synthetase [glutamine-hydrolyzing];Asns;ortholog** |  |  |  | | **Wars** | **Tryptophanyl-tRNA synthetase, cytoplasmic;Wars;ortholog** |  |  |  | | **Trib3** | **Tribbles homolog 3;Trib3;ortholog** |  | ***** |  | |  |  |  |  |  | | **GTP binding proteins** |  |  |  |  | | **Mras** | **Ras-related protein M-Ras;Mras;ortholog** |  |  |  | | **Rtkn** | **Rhotekin;Rtkn;ortholog** |  | ***** | ***** | | **Tubb2a** | **Tubulin beta-2A chain;Tubb2a;ortholog** | ***** |  |  | |  |  |  |  |  | | **Ubiquitin pathway** |  |  |  |  | | **Fbxo7** | **F-box only protein 7;Fbxo7;ortholog** |  | ***** | ***** | | **Wwp2** | **NEDD4-like E3 ubiquitin-protein ligase WWP2;Wwp2;ortholog** | ***** | ***** | ***** | | **Rnf144a** | **Probable E3 ubiquitin-protein ligase RNF144A;Rnf144a;ortholog** |  | ***** | ***** | | **Ube2e3** | **Ubiquitin-conjugating enzyme E2 E3;Ube2e3;ortholog** |  |  |  | |  |  |  |  |  | | **Not classified** |  |  |  |  | | **Hr** | **Protein hairless;Hr;ortholog** |  | ***** |  | | **Lars** | **Leucyl-tRNA synthetase, cytoplasmic;Lars;ortholog** |  |  |  | | **Tsc22d3** | **TSC22 domain family protein 3;Tsc22d3;ortholog** |  |  |  | | **Pde4dip** | **Myomegalin;Pde4dip;ortholog** |  | ***** |  | | **Eif1ad** | **Probable RNA-binding protein EIF1AD;Eif1ad;ortholog** |  |  |  | | **Hip1r** | **Huntingtin-interacting protein 1-related protein;Hip1r;ortholog** | ***** | ***** |  | | **Fry** | **Fry;Fry;ortholog** | ***** | ***** | ***** | | **Scarb2** | **Lysosome membrane protein 2;Scarb2;ortholog** |  |  |  | | **Shf** | **SH2 domain-containing adapter protein F;Shf;ortholog** |  |  |  | | **Tprgl** | **Tumor protein p63-regulated gene 1-like protein;Tprg1l;ortholog** |  | ***** | ***** | | **Mtss1l** | **MTSS1-like protein;Mtss1l;ortholog** |  | ***** | ***** | | **Vps54** | **Vacuolar protein sorting-associated protein 54;Vps54;ortholog** |  |  |  | | **Ddit3** | **DNA damage-inducible transcript 3;Ddit3;ortholog** |  |  |  | | **Spats2l** | **SPATS2-like protein;Spats2l;ortholog** |  | ***** |  | | **Trim47** | **Tripartite motif-containing protein 47;Trim47;ortholog** |  | ***** |  | | **Dnttip1** | **Deoxynucleotidyltransferase terminal-interacting protein 1;Dnttip1;ortholog** |  | ***** |  | | **Chadl** | **Chadl;Chadl;ortholog** | ***** | ***** | ***** | | **WSCD2** | **WSC domain containing 2** |  | ***** |  | | **Susd5** | **sushi domain containing 5** | ***** | ***** | ***** | | **Fam89a** | **hypothetical protein LOC100047808; similar to mammary tumor virus receptor 2 isoform-like** |  | ***** | ***** | | **Apeg3** | **paternally expressed 3; antisense transcript gene of Peg3** | ***** |  |  | | **Klk10** | **kallikrein related-peptidase 10** |  |  |  | | **B230206H07Rik** | **RIKEN cDNA B230206H07 gene** |  | ***** | ***** | | **Prr5** | **Protor-1;Protor1;ortholog** |  |  | ***** | | **Gadd45a** | **Growth arrest and DNA-damage-inducible protein GADD45 alpha;Gadd45a;ortholog** |  |  |  | | **Ppp1r1b** | **Protein phosphatase 1 regulatory subunit 1B;Ppp1r1b;ortholog** |  | ***** |  | | **Tnpo3** | **Transportin-3;Tnpo3;ortholog** |  |  |  | | **Peg10** | **Retrotransposon-derived protein PEG10;Peg10;ortholog** |  |  |  | | **Sema6a** | **Semaphorin-6A;Sema6a;ortholog** |  |  | ***** | | **Vps54** | **Vacuolar protein sorting-associated protein 54;Vps54;ortholog** |  |  |  | | **Myo6** | **Myosin-VI;Myo6;ortholog** |  |  |  | | **Ugp2** | **UTP--glucose-1-phosphate uridylyltransferase;Ugp2;ortholog** |  |  |  | | **G0s2** | **Putative lymphocyte G0/G1 switch protein 2;G0s2;ortholog** |  |  |  | | **Peg3** | **Paternally-expressed gene 3 protein;Peg3;ortholog** | ***** |  |  | | **Sobp** | **Sine oculis-binding protein homolog;Sobp;ortholog** |  | ***** | ***** | | **Mid2** | **Midline-2;Mid2;ortholog** |  |  |  | | **Cpm** | **Carboxypeptidase M;Cpm;ortholog** |  | ***** | ***** | | **Dhrs7** | **Dehydrogenase/reductase SDR family member 7;Dhrs7;ortholog** |  |  |  | | **Ppp2ca** | **Serine/threonine-protein phosphatase 2A catalytic subunit alpha isoform;Ppp2ca;ortholog** |  | ***** |  | | **Capn6** | **Calpain6** |  |  |  | | **Phyh** | **phytanoyl-CoA hydroxylase** | ***** | ***** |  | | **Ogfod1** | **2-oxo 1glutarate and iron-dependent oxygenase domain containing** |  |  |  | |
| --- | --- | --- | --- | --- | --- | --- | --- | --- | --- | --- | --- | --- | --- | --- | --- | --- | --- | --- | --- | --- | --- | --- | --- | --- | --- | --- | --- | --- | --- | --- | --- | --- | --- | --- | --- | --- | --- | --- | --- | --- | --- | --- | --- | --- | --- | --- | --- | --- | --- | --- | --- | --- | --- | --- | --- | --- | --- | --- | --- | --- | --- | --- | --- | --- | --- | --- | --- | --- | --- | --- | --- | --- | --- | --- | --- | --- | --- | --- | --- | --- | --- | --- | --- | --- | --- | --- | --- | --- | --- | --- | --- | --- | --- | --- | --- | --- | --- | --- | --- | --- | --- | --- | --- | --- | --- | --- | --- | --- | --- | --- | --- | --- | --- | --- | --- | --- | --- | --- | --- | --- | --- | --- | --- | --- | --- | --- | --- | --- | --- | --- | --- | --- | --- | --- | --- | --- | --- | --- | --- | --- | --- | --- | --- | --- | --- | --- | --- | --- | --- | --- | --- | --- | --- | --- | --- | --- | --- | --- | --- | --- | --- | --- | --- | --- | --- | --- | --- | --- | --- | --- | --- | --- | --- | --- | --- | --- | --- | --- | --- | --- | --- | --- | --- | --- | --- | --- | --- | --- | --- | --- | --- | --- | --- | --- | --- | --- | --- | --- | --- | --- | --- | --- | --- | --- | --- | --- | --- | --- | --- | --- | --- | --- | --- | --- | --- | --- | --- | --- | --- | --- | --- | --- | --- | --- | --- | --- | --- | --- | --- | --- | --- | --- | --- | --- | --- | --- | --- | --- | --- | --- | --- | --- | --- | --- | --- | --- | --- | --- | --- | --- | --- | --- | --- | --- | --- | --- | --- | --- | --- | --- | --- | --- | --- | --- | --- | --- | --- | --- | --- | --- | --- | --- | --- | --- | --- | --- | --- | --- | --- | --- | --- | --- | --- | --- | --- | --- | --- | --- | --- | --- | --- | --- | --- | --- | --- | --- | --- | --- | --- | --- | --- | --- | --- | --- | --- | --- | --- | --- | --- | --- | --- | --- | --- | --- | --- | --- | --- | --- | --- | --- | --- | --- | --- | --- | --- | --- | --- | --- | --- | --- | --- | --- | --- | --- | --- | --- | --- | --- | --- | --- | --- | --- | --- | --- | --- | --- | --- | --- | --- | --- | --- | --- | --- | --- | --- | --- | --- | --- | --- | --- | --- | --- | --- | --- | --- | --- | --- | --- | --- | --- | --- | --- | --- | --- | --- | --- | --- | --- | --- | --- | --- | --- | --- | --- | --- | --- | --- | --- | --- | --- | --- | --- | --- | --- | --- | --- | --- | --- | --- | --- | --- | --- | --- | --- | --- | --- | --- | --- | --- | --- | --- | --- | --- | --- | --- | --- | --- | --- | --- | --- | --- | --- | --- | --- | --- | --- | --- | --- | --- | --- | --- | --- | --- | --- | --- | --- | --- | --- | --- | --- | --- | --- | --- | --- | --- | --- | --- | --- | --- | --- | --- | --- | --- | --- | --- | --- | --- | --- | --- | --- | --- | --- | --- | --- | --- | --- | --- | --- | --- | --- | --- | --- | --- | --- | --- | --- | --- | --- | --- | --- | --- | --- | --- | --- | --- | --- | --- | --- | --- | --- | --- | --- | --- | --- | --- | --- | --- | --- | --- | --- | --- | --- | --- | --- | --- | --- | --- | --- | --- | --- | --- | --- | --- | --- | --- | --- | --- | --- | --- | --- | --- | --- | --- | --- | --- | --- | --- | --- | --- | --- | --- | --- | --- | --- | --- | --- | --- | --- | --- | --- | --- | --- | --- | --- | --- | --- | --- | --- | --- | --- | --- | --- | --- | --- | --- | --- | --- | --- | --- | --- | --- | --- | --- | --- | --- | --- | --- | --- | --- | --- | --- | --- | --- | --- | --- | --- | --- | --- | --- | --- | --- | --- | --- | --- | --- | --- | --- | --- | --- | --- | --- | --- | --- | --- | --- | --- | --- | --- | --- | --- | --- | --- | --- | --- | --- | --- | --- | --- | --- | --- | --- | --- | --- | --- | --- | --- | --- | --- | --- | --- | --- | --- | --- | --- | --- | --- | --- | --- | --- | --- | --- | --- | --- | --- | --- | --- | --- | --- | --- | --- | --- | --- | --- | --- | --- | --- | --- | --- | --- | --- | --- | --- | --- | --- | --- | --- | --- | --- | --- | --- | --- | --- | --- | --- | --- | --- | --- | --- | --- | --- | --- | --- | --- | --- | --- | --- | --- | --- | --- | --- | --- | --- | --- | --- | --- | --- | --- | --- | --- | --- | --- | --- | --- | --- | --- | --- | --- | --- | --- | --- | --- | --- | --- | --- | --- | --- | --- | --- | --- | --- | --- | --- | --- | --- | --- | --- | --- | --- | --- | --- | --- | --- | --- | --- | --- | --- | --- | --- | --- | --- | --- | --- | --- | --- | --- | --- | --- | --- | --- | --- | --- | --- | --- | --- | --- | --- | --- | --- | --- | --- | --- | --- | --- | --- | --- | --- | --- | --- | --- | --- | --- | --- | --- | --- | --- | --- | --- | --- | --- | --- | --- | --- | --- | --- | --- | --- | --- | --- | --- | --- | --- | --- | --- | --- | --- | --- | --- | --- | --- | --- | --- | --- | --- | --- | --- | --- | --- | --- | --- | --- | --- | --- | --- | --- | --- | --- | --- | --- | --- | --- | --- | --- | --- | --- | --- | --- | --- | --- | --- | --- | --- | --- | --- | --- | --- | --- | --- | --- | --- | --- | --- | --- | --- | --- | --- | --- | --- | --- | --- | --- | --- | --- | --- | --- | --- | --- | --- | --- | --- | --- | --- | --- | --- | --- | --- | --- | --- | --- | --- | --- | --- | --- | --- | --- | --- | --- | --- | --- | --- | --- | --- | --- | --- | --- | --- | --- | --- | --- | --- | --- | --- | --- | --- | --- | --- | --- | --- | --- | --- | --- | --- | --- | --- | --- | --- | --- | --- | --- | --- | --- | --- | --- | --- | --- | --- | --- | --- | --- | --- | --- | --- | --- | --- | --- | --- | --- | --- | --- | --- | --- | --- | --- | --- | --- | --- | --- | --- | --- | --- | --- | --- | --- | --- | --- | --- | --- | --- | --- | --- | --- | --- | --- | --- | --- | --- | --- | --- | --- | --- | --- | --- | --- | --- | --- | --- | --- | --- | --- | --- | --- | --- | --- | --- | --- | --- | --- | --- | --- | --- | --- | --- | --- | --- | --- | --- | --- | --- | --- | --- | --- | --- | --- | --- | --- | --- | --- | --- | --- | --- | --- | --- | --- | --- | --- | --- | --- | --- | --- | --- | --- | --- | --- | --- | --- | --- | --- | --- | --- | --- | --- | --- | --- | --- | --- | --- | --- | --- | --- | --- | --- | --- | --- | --- | --- | --- | --- | --- | --- | --- | --- | --- | --- | --- | --- | --- | --- | --- | --- | --- | --- | --- | --- | --- | --- | --- | --- | --- | --- | --- | --- | --- | --- | --- | --- | --- | --- | --- | --- | --- | --- | --- | --- | --- | --- | --- | --- | --- | --- | --- | --- | --- | --- | --- | --- | --- | --- | --- | --- | --- | --- | --- | --- | --- | --- | --- | --- | --- | --- | --- | --- | --- | --- | --- | --- | --- | --- | --- | --- | --- | --- | --- | --- | --- | --- | --- | --- | --- | --- | --- | --- | --- | --- | --- | --- | --- | --- | --- | --- | --- | --- | --- | --- | --- | --- | --- | --- | --- | --- | --- | --- | --- | --- | --- | --- | --- | --- | --- | --- | --- | --- | --- | --- | --- | --- | --- | --- | --- | --- | --- | --- | --- | --- | --- | --- | --- | --- | --- | --- | --- | --- |

**This table also shows the genes for which there is some evidence of association with human disease according to the University of Copenhagen Disease Data Base (http//www.disease.jensenlab.org).**
