# Supplementary material for: SOX9 Regulates Multiple Genes in Chondrocytes, Including Genes Encoding ECM Proteins, ECM Modification Enzymes, Receptors, and Transporters
Source: PLoS One. 2014 Sep 17;9(9):e107577. doi: 10.1371/journal.pone.0107577 (PMC4168005; doi:10.1371/journal.pone.0107577)
Supplement: Table S4 — Genes with increased expression by more than 8-fold after removal of Sox9. (DOC) [file pone.0107577.s007.doc]

**Table S4. Genes with increased expression by more than 8-fold after removal of *Sox9***

| **Gene ID** | **Name** | **fold increase** |
| --- | --- | --- |
| *Itga11* | integrin, alpha11 | 8.1 |
| *Adamts4* | Adam metallopeptidase with thrombospondin type1, motif, 4 | 9.6 |
| *AI593442* | expressed sequence AI593442 | 9.6 |
| *Mt1* | metallothionein 1 | 12.7 |
| *Aqp1* | aquaporin 1 | 15.6 |
| *Krt19* | keratin 19 | 51.3 |
